# Effects of vitamin D in pregnancy on maternal and offspring health-related outcomes: An umbrella review of systematic review and meta-analyses

**DOI:** 10.1038/s41387-024-00296-0

**Published:** 2024-05-30

**Authors:** Mei-Chun Chien, Chueh-Yi Huang, Jie-Huei Wang, Chia-Lung Shih, Pensee Wu

**Affiliations:** 1 Department of Rehabilitation, Ditmanson Medical Foundation Chia-Yi Christian Hospital, Chia-Yi City, Taiwan; 2 Department of Obstetrics and Gynecology, Ditmanson Medical Foundation Chia-Yi Christian Hospital, Chia-Yi City, Taiwan; 3 Department of Mathematics, National Chung Cheng University, Chia-Yi, Taiwan; 4 Clinical Research Center, Ditmanson Medical Foundation Chia-Yi Christian Hospital, Chia-Yi City, Taiwan; 5 School of Medicine, Keele University, Staffordshire, UK; 6 Academic Department of Obstetrics and Gynaecology, University Hospital of North Midlands, Stoke-on-Trent, UK; 7 Department of Obstetrics and Gynecology, College of Medicine, National Cheng Kung University, Tainan, Taiwan

**Keywords:** Diseases, Health care

## Abstract

**Background:**

Vitamin D deficiency has been linked with several adverse maternal and fetal outcomes.

**Objective:**

To summarize systematic reviews and meta-analyses evaluating the effects of vitamin D deficiency and of vitamin D supplementation in pregnancy on maternal and offspring health-related outcomes.

**Methods:**

Prior to conducting this umbrella review, we registered the protocol in PROSPERO (CRD42022368003). We conducted searches in PubMed, Embase, and Cochrane Library for systematic reviews and meta-analyses on vitamin D in pregnancy, from database inception to October 2, 2023. All outcomes related to vitamin D in pregnancy obtained from the systematic reviews and meta-analyses were extracted. Data Extraction: Two reviewers independently chose studies and collected information on health outcomes. The quality of the included articles’ methodology was assessed using AMSTAR 2 (A Measurement Tool to Assess Systematic Reviews–2).

**Results:**

We identified 16 eligible systematic reviews and meta-analyses, which included 250,569 women. Our results demonstrated that vitamin D deficiency in pregnancy is associated with increased risk of preterm birth, small-for gestational age/low birth weight infants, recurrent miscarriage, bacterial vaginosis and gestational diabetes mellitus. Vitamin D supplementation in pregnancy increases birth weight, and reduces the risk of maternal pre-eclampsia, miscarriage, and vitamin D deficiency, fetal or neonatal mortality, as well as attention-deficit hyperactivity disorder, and autism spectrum disorder in childhood. In women with gestational diabetes mellitus, vitamin D supplementation in pregnancy can reduce the risk of maternal hyperbilirubinemia, polyhydramnios, macrosomia, fetal distress, and neonatal hospitalization.

**Conclusion:**

Due to the association with adverse maternal and offspring health outcomes, we recommend the vitamin D status in pregnancy should be monitored, particularly in women at high risk of vitamin D deficiency. It is suggested that pregnant women take a dose of >400 IU/day of vitamin D supplementation during pregnancy to prevent certain adverse outcomes.

## Background

Vitamin D is a fat soluble steroid hormone important in the homeostasis of multiple organs [1]. In addition to regulating phosphorus and calcium levels and promoting bone mineralization, it has extraskeletal functions in cardiovascular, metabolic, respiratory and immune systems [2–6]. Vitamin D deficiency is reemerging with high-risk groups including people with little sunshine exposure, people with darker skin and pregnant women [7].

During pregnancy, the body undergoes significant physiological change, including requirements and metabolism of vitamin D. Vitamin D deficiency in pregnancy is a common problem worldwide. The prevalence varies between 5% to 90% depending on the country [8–10]. Vitamin D deficiency has been linked with several adverse maternal and fetal outcomes [11–13]. For example, hypertensive disorders of pregnancy, gestational diabetes mellitus (GDM), preterm birth, and low birthweight [14].

To date, several meta-analyses have been conducted to investigate the associations between vitamin D status during pregnancy and maternal and offspring outcomes [15, 16]. Additionally, many meta-analyses have examined the impact of vitamin D supplementation during pregnancy on these outcomes [17, 18]. An umbrella review employs systematic review methods to collect the current high-level evidence from meta-analyses on a specific topic [19]. However, the evidence regarding to vitamin D status during pregnancy associated with maternal and offspring outcomes has not been well-organized using an umbrella review.

To provide clinicians, researchers and policy makers an overview of the complete body of evidence, we synthesized the findings, quality and certainty of systematic reviews and meta-analyses evaluating the effects of vitamin D deficiency and of vitamin D supplementation in pregnancy on maternal and offspring health-related outcomes.

## Method

### Protocol and registration

Prior to conducting this umbrella review, we registered the protocol in PROSPERO (CRD42022368003) (Tables S1 and S2).

### Search methods

Three electronic databases (PubMed, Embase, and Cochrane Library) were searched for relevant systematic reviews and meta-analyses investigating the effects of vitamin D in pregnancy on maternal and infant health from inception to October 2 2023. The complete search strategy is displayed in Table S3. Initial deduplication was performed using EndNote 20, followed by title/abstract screening. Finally, full-text screening was performed to determine whether the remaining meta-analyses met our inclusion criteria. Two authors (C.L.S and P.W.) independently conducted title/abstract screening. Any disagreement was resolved through discussion. In addition, we manually searched the reference lists of the included systematic reviews and meta-analyses or related reviews to identify potential meta-analyses for inclusion.

### Eligibility criteria

We used the PECOS (P: population, E: exposure, C: comparison, O: outcomes, and S: study design) framework to determine the inclusion criteria. The criteria were as follows: (1) population: pregnant women; (2) exposure: intake of vitamin D or measured vitamin D levels; (3) comparison: authors defined either a low level of vitamin D supplementation, placebo, no supplementation, or low measured vitamin D levels; (4) outcomes: all health-related outcomes; (5) study design: meta-analyses of randomized controlled or observational studies. If any meta-analyses overlapped with more than one similar outcome, we included the newest meta-analysis and if they were published in the same year, we included the meta-analysis with the largest number of studies in our review. The exclusion criteria were as follows: (1) conference abstract; (2) animal study; (3) meta-analyses not from systematic reviews; and (3) meta-analyses that were not published in English or Chinese.

### Data extraction

We extracted the following information from the included meta-analysis: name of first author, publication year, type of clinical outcome, study design, number of studies in each meta-analysis, total sample size, effect sizes (OR: odds ratio, RR: relative risk, MD: mean difference, and SMD: standardized mean difference). One author (C.L.S.) conducted the data extraction, and another author (P.W.) checked the data. Any disagreement was resolved through consensus or by consulting a third reviewer (C.Y.H.).

The pooled estimates with the 95% confidence interval (CI) of clinical outcomes were extracted from each included meta-analysis. The corresponding evidence of heterogeneity (*I*^2^ value) was also extracted from the meta-analysis. Moreover, if available, publication bias assessed by Egger’s test or Begg’s test was obtained from the included meta-analysis.

### Assessment of quality and evidence

A Measurement Tool to Assess Systematic Reviews 2 (AMSTAR 2) was utilized to assess each meta-analysis’s metrological quality [20]. The instrument has the capability to categorize every meta-analysis’s quality into four groups: high (containing no or one non-critical weakness), moderate (containing multiple non-critical weaknesses), low (containing one critical flaw from protocol registration, adequate literature search, rationale for excluding studies, risk of bias evaluated for every included study, suitable meta-analytical approach, taking into account risk of bias when interpreting review results, and evaluation of publication bias), and critically low (containing multiple critical flaws). Moreover, we used a modified criteria to evaluate the quality of evidence for the meta-analysis [21]. The criteria adopted include the number of included studies, heterogeneity, and differences in results between the two study designs (randomized controlled trials (RCTs) and observational studies) in the same meta-analysis. In general, the quality of evidence is classified into four levels, from the highest (level 1: convincing) to the lowest (level 4: limited/contrasting).

## Results

### Search results

**Fig. 1:**
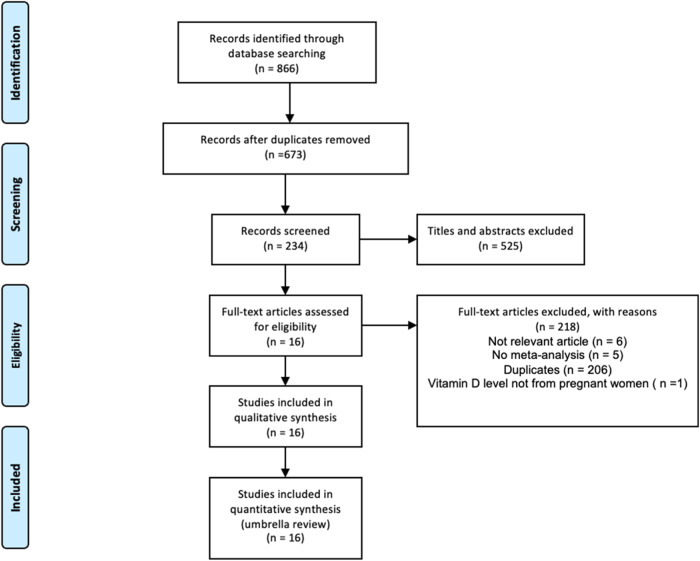
Selection process of relevant articles. Three electronic databases were searched for relevant articles. Initial deduplication was performed using EndNote 20, followed by title/abstract screening. Finally, full-text screening was performed to determine whether the remaining meta-analyses met our inclusion criteria.

The process of literature search and study selection is shown in Fig. 1. A total of 866 meta-analyses were initially identified from the searches. Following deduplication, 673 meta-analyses remained, which underwent title and abstract screening with 123 meta-analyses selected for full-text screening. Finally, 16 meta-analyses met the inclusion criteria for this umbrella review [15–18, 22–34].

### Study characteristics

Total number of studies included in each included meta-analysis ranged from 4 to 54. The total number of participants for each included meta-analysis ranged from 1,465 to 67,484. Among them, 9 meta-analyses focused on observational studies and reported 16 specific outcomes. The other 7 meta-analyses focused on randomized controlled trials. Of those, 31 specific outcomes were obtained from pregnant women, while 18 specific outcomes were obtained from pregnant women with GDM (Table 1).

**Table 1:** Characteristics of the included meta-analyses.

OBS	First author	Publication year	Study design	No. of studies in MA	No of sample size (individual)	Outcomes	AMSTAR 2
1	Tirani [15]	2023	Observation	5	9125	Offspring autism spectrum disorder and offspring attention-deficit hyperactivity disorder	Critically low
2	Wu [17]	2023	RCT	20	1682	25(OH)D, total cholesterol, low-density lipoprotein cholesterol, high-density lipoprotein cholesterol, triglycerides, preterm birth, hyperbilirubinemia, and neonatal hospitalization	Critically low
3	Rouhani [34]	2023	Observation	32	76,394	Pre-eclampsia	Critically low
4	Liu [22]	2022	RCT	42	11,082	Fetal or neonatal mortality, preterm birth, birth length, birth head circumference, and birth weight	Critically low
5	Chen [23]	2022	Observation	14	3029	Recurrent miscarriage	Critically low
6	Luo [25]	2022	RCT	23	5390	Femur length in the third trimester, humeral length in the third trimester, neonatal WB-BMC, neonatal WB-BMD, and neonatal WB-BA	Critically low
7	Luo [26]	2022	RCT	7	3737	Asthma or wheeze, eczema, allergic rhinitis, and lower respiratory tract infection for infants	Critically low
8	Ma [16]	2022	Observation	13	4793	Bacterial vaginosis	Critically low
9	Motamed [18]	2022	RCT	17	1465	25(OH)D, hs-CRP, total antioxidant capacity, malondialdehyde, and glutathione	Critically low
10	Irwinda [27]	2022	RCT	27	7567	Pre-eclampsia, gestational diabetes mellitus, preterm birth, and birth weight	Critically low
11	Fatima [28]	2022	Observation	44	37,838	Gestational diabetes mellitus	Critically low
12	Tamblyn [29]	2022	Observation	10	7663	Miscarriage risk	Critically low
13	Fang [30]	2021	Observation	16	8403	Low birth weight and birth weight	Critically low
14	Wang [31]	2021	RCT	19	1550	Fasting plasma glucose, fasting insulin level, HOMA-IR, Caesarean section rate, maternal hospitalization rate, postpartum hemorrhage, hyperbilirubinemia, macrosomia, polyhydramnios, fetal distress, preterm birth, and hypoglycemia	Critically low
15	Tous [32]	2020	Observation	54	67,484	Birth length, birth head circumference, birth weight, preterm birth, and small-for-gestational-age	Critically low
16	Kang [33]	2020	Observation	4	3367	25(OH)D for pregnant women	Critically low

*RCT* randomized controlled trial, *MA* meta-analysis, *AMSTAR 2* A Measurement Tool to Assess Systematic Reviews 2, *25(OH)D* 25-hydroxyvitamin D, *WB-BMC* whole body bone mineral content, *WB-BMD* whole body bone mineral density, *WB-BA* whole body bone area, *hs-CRP* high sensitivity C-reactive protein, *HOMA-IR* homeostasis model assessment of insulin resistance.

### Assessment of quality and evidence

The metrological quality of each meta-analysis was assessed using AMSTAR 2. All meta-analyses received a grade of “critically low” because more than one critical flaw was detected for each meta-analysis (Table 1). The quality of evidence of the specific outcomes which demonstrated statistically significant difference was assessed. Among the 16 specific outcomes derived from observational studies, most of them (13/16) were positively correlated with vitamin D level during pregnancy (Table S4). Eleven out of these twelve outcomes were graded as “possible” (level 3) due to high heterogeneity (*I*^2^ > 50%), while two were graded as “probable” (level 2) due to low heterogeneity. Among the 31 specific outcomes derived from RCTs, only a few outcomes (11/31) were positively correlated with vitamin D supplementation (Table S5). Nine of these eleven outcomes were graded as “convincing” (level 1) but two of them were graded as “limited” (level 4) due to the small number of meta-analyses with these outcomes (*n* ≤ 3) (Table S6).

### Vitamin D in pregnancy related to health outcomes in observational studies

For maternal outcomes, a low level of vitamin D ( < 50 nmol/L), significantly increased the risk of preterm birth (OR = 1.28; 95% CI = 1.08–1.52; number of studies = 21; Table 2A) [32], miscarriage (OR = 1.60; 95% CI = 1.11–2.30; number of studies = 6; Table 2A) [29], and small-for-gestational-age infants (OR = 1.43; 95% CI = 1.08–1.91; number of studies = 19; Table 2B) [32]. Vitamin D deficiency also increased the risk of GDM (OR = 1.38; 95% CI = 1.22 – 1.57; number of studies = 31) [28], recurrent miscarriage (OR = 4.02; 95% CI = 2.23–7.25; number of studies = 12) [23], and bacterial vaginosis (OR = 1.54; 95% CI = 1.25–1.91; number of studies = 14) (Table 2A) [16]. The highest level of vitamin D had a significant lower risk of preeclampsia compared with the lowest level (RR = 0.68; 95% CI = 0.55–0.85; number of studies = 29; Table 2A) [34].

**Table 2:** Summary of meta-analyses of observational studies on vitamin D in pregnancy. (A) Maternal health. (B) Offspring health.

(A)										
Outcomes	First author	Year	No. of studies in MA	Comparator (vitamin D supplementation or level)	Effects model	Metric of MA	Effect size	95% CI	*I*^*2*^ *(%)*	Publication bias
Preterm birth	Tous	2020	11	low level < 30 vs high level ≥ 30 (nmol/L)	random-effect	OR	1.16	(0.83 ~ 1.62)	68	NA
Preterm birth	Tous	2020	21	low level < 50 vs high level ≥ 50 (nmol/L)	random-effect	OR	1.28	(1.08 ~ 1.52)*	61	NA
Preterm birth	Tous	2020	10	low vit D < 75 vs high vit D ≥ 75 (nmol/L)	random-effect	OR	1.18	(0.91 ~ 1.54)	72	NA
Gestational diabetes mellitus	Fatima	2022	31	vitamin D deficiency vs normal	random-effect	OR	1.38	(1.22 ~ 1.57)*	49	NA
Pre-eclampsia	Rouhani	2023	29	highest vs lowest circulating 25(OH)D levels	random-effect	RR	0.68	(0.55 ~ 0.85)*	98	0.65
Recurrent miscarriage	Chen	2022	12	vitamin D deficiency vs normal	random-effect	OR	4.02	(2.23 ~ 7.25)*	82	NA
Miscarriage risk	Tamblyn	2022	4	low level <50 vs high level ≥ 75 (nmol/L)	random-effect	OR	1.94	(1.25 ~ 3.02)*	18	NA
Miscarriage risk	Tamblyn	2022	6	low level ≤ 75 vs high level > 75 (nmol/L)	random-effect	OR	1.60	(1.11 ~ 2.30)*	35	NA
Bacterial vaginosis	Ma	2022	14	vitamin D deficiency vs normal	random-effect	OR	1.54	(1.25 ~ 1.91)*	85	0.005
25(OH)D	Kang	2020	8	type 1 diabetes in childhood vs normal	random-effect	MD	-2.54	(-4.65 ~ -0.44)*	52	0.04
(**B**)										
Birth length	Tous	2020	4	low level < 30 vs high level ≥ 30 (nmol/L)	random-effect	MD	-0.28 cm	(-0.66 ~ 0.10)	69	NA
Birth length	Tous	2020	7	low level < 50 vs high level ≥ 50 (nmol/L)	random-effect	MD	0.12 cm	(-0.09 ~ 0.33)	63	NA
Low birth weight (< 2500 g)	Fang	2021	8	low level < 50 vs high level ≥ 50 (nmol/L)	random-effect	OR	2.39	(1.25 ~ 4.57)*	81	NA
Birth weight	Tous	2020	15	low level < 30 vs high level ≥ 30 (nmol/L)	random-effect	MD	-87.82 g	(-119.73 ~ -55.91)*	58	NA
Birth weight	Tous	2020	13	low level < 50 vs high level ≥ 50 (nmol/L)	random-effect	MD	-19.27 g	(-63.34 ~ 24.80)	84	NA
Birth weight	Tous	2020	5	low level < 75 vs high level ≥75 (nmol/L)	random-effect	MD	15.15 g	(-12.73 ~ 43.04)	27	NA
Head circumference	Tous	2020	7	low level < 30 vs high level ≥ 30 (nmol/L)	random-effect	MD	-0.19 cm	(-0.32 ~ -0.06)*	66	NA
Head circumference	Tous	2020	7	low level < 50 vs high level ≥ 50 (nmol/L)	random-effect	MD	-0.47 cm	(-1.11 ~ 0.16)	98	NA
Small-for-gestational-age	Tous	2020	11	low level < 30 vs high level ≥ 30 (nmol/L)	random-effect	OR	1.59	(1.24 ~ 2.03)*	71	NA
Small-for-gestational-age	Tous	2020	19	low level < 50 vs high level ≥ 50 (nmol/L)	random-effect	OR	1.43	(1.08 ~ 1.91)*	89	NA
Small-for-gestational-age	Tous	2020	7	low level < 75 vs high level ≥ 75 (nmol/L)	random-effect	OR	0.98	(0.81 ~ 1.17)	39	NA
Attention-deficit hyperactivity disorder	Tirani	2023	5	highest level vs lowest level	random-effect	OR	0.59	(0.44 ~ 0.81)*	48	NA
Autism spectrum disorder	Tirani	2023	5	highest level vs lowest level	random-effect	OR	0.57	(0.33 ~ 0.99)*	82	NA
Mental development	Tous	2022	9	low level < 50 vs high level ≥ 50 (nmol/L)	random-effect	MD	-1.12 points	(-1.82 ~ -0.42)*	70	NA
Language development	Tous	2022	7	low level < 50 vs high level ≥ 50 (nmol/L)	random-effect	MD	-0.35 points	(-1.00 ~ 0.31)	78	NA
Motor development	Tous	2022	15	low level < 50 vs high level ≥ 50 (nmol/L)	random-effect	MD	-0.06 points	(-0.51 ~ 0.40)	65	NA

*MA* meta-analysis, *MD* mean difference, *OR* odds ratio, *RR* relative risk, *NA* not available, * significant difference, *25(OH)D* 25-hydroxyvitamin D.

For the offspring, a low level of vitamin D ( < 30 nmol/L) significantly reduced birth weight (MD: −87.83; 95% CI: −119.73 ~ −55.91; number of studies = 15; Table 2B) and decreased head circumference compared with a high level of vitamin D (MD = −0.19; 95% CI = −0.32 ~ −0.06; number of studies = 7; Table 2B) [32]. Pregnant women with vitamin D deficiency (<50 nmol/L) had a significantly higher rate of low birth weight (<2500 g) than those without deficiency (OR = 2.39; 95% CI = 1.25 ~ 4.57; number of studies = 8; Table 2B) [30]. A high level of vitamin D ( > 50 nmol/L) significantly decreased the risk of attention-deficit hyperactivity disorder (OR = 0.59; 95% CI = 0.44 ~ 0.81; number of studies = 5; Table 2B) and autism spectrum disorder (OR = 0.57; 95% CI = 0.33 ~ 0.99; number of studies = 5; Table 2B) [15]. Furthermore, a low level of vitamin D ( < 50 nmol/L) significantly decreased the mental development score in the child (MD = −1.12; 95% CI = −1.82 ~ −0.42; number of studies = 9; Table 2B) in comparison to a high level (>50 nmol/L) of vitamin D [32]. Children with type 1 diabetes had significantly lower maternal levels of vitamin D compared to children without type 1 diabetes (MD = −2.54; 95% CI = −4.65 ~ −0.44; number of studies = 8; Table 2A) [33].

### Vitamin D supplementation in pregnancy associated with health-related outcomes from RCTs

High dose vitamin D supplementation (>2000 IU/day) reduced the risk of GDM compared with a low dose supplementation (≤2000 IU/day) (OR = 0.7; 95% CI = 0.51–0.95; number of studies = 7; Table 3) [27], while low dose supplementation (≤ 2000 IU/day) reduced the risk of pre-eclampsia compared with placebo (OR = 0.29; 95% CI = 0.09–0.95; number of studies = 3; Table 3). Vitamin D supplementation (>400 IU/day) increased maternal 25-hydroxyvitamin D levels (SMD = 2.07; 95% CI = 1.51 ~ 2.63; number of studies = 15), total antioxidant capacity (SMD = 2.13; 95% CI = 1.04–3.23; number of studies = 9) and glutathione level (SMD = 4.37; 95% CI = 2.90–5.84; number of studies = 9) [18], while decreased maternal the malondialdehyde level (SMD = −0.46; 95% CI = −0.87 ~ −0.05; number of studies = 9) [18], compared with low dose (≤400 IU/day) or placebo (Table 3).

**Table 3:** Summary of meta-analyses of randomized controlled trials on vitamin D in pregnancy in relation to maternal health.

Outcomes	First author	Year	No. of studies in MA	Comparator (vitamin D supplementation or level)	Effects model	Metric of MA	Effect size	95% CI	*I*^2^ (%)	Publication bias
Preterm birth	Liu	2022	27	high dose (> 400 IU/day) vs low dose (≤ 400 IU/day) or placebo	random-effect	RR	0.938	(0.790 ~ 1.090)	9	NA
Preterm birth	Wu	2023	10	high dose (≥ 400 IU/day) vs placebo	fixed-effect	OR	0.37	(0.22 ~ 0.62)*	0	NA
Preterm birth	Irwinda	2022	6	dose≤ 2000 IU/day vs placebo	random-effect	OR	0.84	(0.46 ~ 1.53)	42	NA
Preterm birth	Irwinda	2022	10	high dose (> 2000 IU/day) vs low dose (≤ 2000 IU/day)	random-effect	OR	1.01	(0.82 ~ 1.26)	0	NA
Pre-eclampsia	Irwinda	2022	3	dose ≤ 2000 (IU/day) vs placebo	random-effect	OR	0.29	(0.09 ~ 0.95)*	0	NA
Pre-eclampsia	Irwinda	2022	8	high dose (> 2000 IU/day) vs low dose (≤2000 IU/day)	random-effect	OR	0.8	(0.51 ~ 1.24)	31	NA
Gestational diabetes mellitus	Irwinda	2022	2	dose ⩽ 2000 IU/day vs placebo	random-effect	OR	0.92	(0.59 ~ 1.42)	0	
Gestational diabetes mellitus	Irwinda	2022	7	high dose (> 2000 IU/day) vs low dose (≤2000 IU/day)	random-effect	OR	0.7	(0.51 ~ 0.95)*	0	NA
25(OH)D	Wu	2023	9	high dose (≥400 IU/day) vs placebo	random-effect	SMD	4.07	(2.73 ~ 5.41)*	97	NA
25(OH)D	Motamed	2022	15	high dose (> 400 IU/day) vs low dose (≤ 400 IU/day) or placebo	random-effect	SMD	2.07	(1.51 ~ 2.63)*	94	<0.001
25(OH)D on cord blood	Motamed	2022	3	high dose (> 400 IU/day) vs low dose (≤ 400 IU/day) or placebo	random-effect	SMD	1.13	(-0.28 ~ 2.54)	96	0.77
Total cholesterol	Wu	2023	7	high dose (≥ 400 IU/day) vs placebo	random-effect	SMD	(-0.67)	(-1.19 ~ -0.14)*	85	NA
Low-density lipoprotein cholesterol	Wu	2023	7	high dose (≥ 400 IU/day) vs placebo	fixed-effect	SMD	(-0.49)	(-0.68 ~ -0.29)*	30	NA
Triglycerides	Wu	2023	6	high dose (≥ 400 IU/day) vs placebo	random-effect	SMD	(-0.59)	(-1.01 ~ -0.17)*	77	NA
High-density lipoprotein cholesterol	Wu	2023	8	high dose (≥ 400 IU/day) vs placebo	fixed-effect	SMD	0.41	(0.23 ~ 0.58)*	0	NA
Caesarean section	Wang	2021	9	supplement vs placebo or nothing	fixed-effect	RR	0.75	(0.63 ~ 0.89)*	43	NA
Hospitalization	Wang	2021	2	supplement vs placebo or nothing	fixed-effect	RR	0.13	(0.02 ~ 0.98)*	0	NA
Postpartum hemorrhage	Wang	2021	2	supplement vs placebo or nothing	fixed-effect	RR	0.47	(0.22 ~ 1.00)	0	NA
Hyperbilirubinemia	Wu	2023	9	high dose (≥ 400 IU/day) vs placebo	fixed-effect	OR	0.38	(0.25 ~ 0.58)*	0	NA
hs-CRP	Motamed	2022	10	high dose (> 400 IU/day) vs low dose (≤ 400 IU/day) or placebo	random-effect	SMD	0.24	(-0.56 ~ 1.04)	95	0.22
Total antioxidant capacity	Motamed	2022	9	high dose (> 400 IU/day) vs low dose (≤ 400 IU/day) or placebo	random-effect	SMD	2.13	(1.04 ~ 3.23)*	97	<0.001
Malondialdehyde	Motamed	2022	6	high dose (> 400 IU/day) vs low dose (≤ 400 IU/day) or placebo	random-effect	SMD	(-0.46)	(-0.87 ~ -0.05)*	75	0.003
Glutathione	Motamed	2022	9	high dose (> 400 IU/day) vs low dose (≤ 400 IU/day) or placebo	random-effect	SMD	4.37	(2.90 ~ 5.84)*	98	<0.001

*MA* meta-analysis, *RR* relative risk, *OR* odds ratio, *SMD* standardized mean difference, *NA* not available, * significant difference, *25(OH)D* 25-hydroxyvitamin D, *hs-CRP* high sensitivityC-reactive protein.

In terms of the offspring, vitamin D supplementation (>400 IU/day) significantly increased the humeral length in the third trimester (MD = 0.13; 95% CI = 0.06–0.21; number of studies = 2) [25] and birth length (MD = 0.269; 95% CI = 0.024–0.514; number of studies = 20) and significantly decreased the risk of fetal or neonatal mortality (RR = 0.69; 95% CI = 0.482–0.985; number of studies = 13) [22], compared to low dose supplementation (≤400 IU/day) or placebo (Table 4). However, high dose supplementation (>400 IU/day) did not affect other outcomes (femur length in the third trimester, macrosomia, birth weight, rate of low birth weight, or head circumference). Furthermore, vitamin D supplementation in pregnancy improved the neonatal vitamin D level (MD = 27.7; 95% CI = 20.5–34.9; number of studies = 25) and decreased the risk of neonatal vitamin D insufficiency (RR = 0.508; 95% CI = 0.384–0.673; number of studies = 12) (Table 4) [22]. However, it did not improve infant bone health and small-for-gestational-age infants (Table 4).

**Table 4:** Summary of meta-analyses of randomized controlled trials on vitamin D in pregnancy in relation to offspring health.

Outcomes	First author	Year	No. of studies in MA	Comparator (vitamin D supplementation or level)	Effects model	Metric of MA	Effect size	95% CI	*I*^2^ (%)	Publication bias
Birth length	Liu	2022	20	high dose (> 400 IU/day) vs low dose (≤ 400 IU/day) or placebo	random-effect	MD	0.269 cm	(0.024–0.514)*	63	NA
Low birth weight rate	Liu	2022	10	high dose (> 400 IU/day) vs low dose (≤ 400 IU/day) or placebo	random-effect	RR	0.902	(0.655–1.242)	32	NA
Birth weight	Liu	2022	33	high dose (> 400 IU/day) vs low dose (≤ 400 IU/day) or placebo	random-effect	MD	37.07 g	(-9.669–83.801)	77	NA
Head circumference	Liu	2022	18	high dose (> 400 IU/day) vs low dose (≤ 400 IU/day) or placebo	random-effect	MD	0.149 cm	(-0.016 ~ 0.315)	68	NA
Small-for-gestational-age	Liu	2022	14	high dose (> 400 IU/day) vs low dose (≤ 400 IU/day) or placebo	random-effect	RR	0.836	(0.633 ~ 1.104)	20	NA
Macrosomia	Liu	2022	3	high dose (> 400 IU/day) vs low dose (≤ 400 IU/day) or placebo	random-effect	RR	1.097	(0.600 ~ 2.007)	0	NA
Femur length in the third trimester	Luo^a^	2022	2	high dose (> 400 IU/day) vs low dose (≤ 400 IU/day) or placebo	random-effect	MD	0.16	(-0.07 ~ 0.40)	60	NA
humeral length in the third trimester	Luo^a^	2022	2	high dose (> 400 IU/day) vs low dose (≤ 400 IU/day) or placebo	fixed-effect	MD	0.13	(0.06 ~ 0.21)*	0	NA
Congenital anomalies	Liu	2022	4	high dose (> 400 IU/day) vs low dose (≤ 400 IU/day) or placebo	random-effect	RR	0.759	(0.439 ~ 1.314)	56	NA
1-min Apgar score	Liu	2022	4	high dose (> 400 IU/day) vs low dose (≤ 400 IU/day) or placebo	random-effect	MD	0.093	(-0.004 ~ 0.190)	20.5	NA
5-min Apgar score	Liu	2022	4	high dose (> 400 IU/day) vs low dose (≤ 400 IU/day) or placebo	random-effect	MD	0.041	(-0.051 ~ 0.133)	43.6	NA
Newborn NICU admission or hospitalization	Liu	2022	5	high dose (> 400 IU/day) vs low dose (≤ 400 IU/day) or placebo	random-effect	RR	1.00	(0.830 ~ 1.205)	0	NA
Asthma or wheeze	Luo	2022	4	high dose (> 400 IU/day) vs low dose (≤ 400 IU/day) or placebo	fixed-effect	RR	1.01	(0.81 ~ 1.26)	47	NA
Eczema	Luo	2022	3	high dose (> 400 IU/day) vs low dose (≤ 400 IU/day) or placebo	fixed-effect	RR	0.95	(0.80 ~ 1.13)	0	NA
Allergic rhinitis	Luo	2022	3	high dose (> 400 IU/day) vs low dose (≤ 400 IU/day) or placebo	fixed-effect	RR	0.93	(0.78 ~ 1.11)	47	NA
Fetal or neonatal mortality	Liu	2022	13	high dose (> 400 IU/day) vs low dose (≤ 400 IU/day) or placebo	random-effect	RR	0.69	(0.482 ~ 0.985)*	0	NA
Lower respiratory tract infection	Luo	2022	3	high dose (> 400 IU/day) vs low dose (≤ 400 IU/day) or placebo	fixed-effect	RR	0.97	(0.85 ~ 1.11)	0	NA
Hyperbilirubinemia	Wang	2021	7	supplement vs placebo or nothing	fixed-effect	RR	0.47	(0.33 ~ 0.67)*	0	NA
Macrosomia	Wang	2021	6	supplement vs placebo or nothing	fixed-effect	RR	0.58	(0.38 ~ 0.89)*	0	NA
Fetal distress	Wang	2021	2	supplement vs placebo or nothing	fixed-effect	RR	0.46	(0.24 ~ 0.90)*	0	NA
Polyhydramnios	Wang	2021	4	supplement vs placebo or nothing	fixed-effect	RR	0.42	(0.24 ~ 0.72)*	0	NA
Hypoglycemia	Wang	2021	4	supplement vs placebo or nothing	fixed-effect	RR	0.82	(0.52 ~ 1.29)	0	NA
Hospitalization	Wu	2023	4	high dose (⩾ 400 IU/day) vs placebo	fixed-effect	OR	0.29	(0.16 ~ 0.53)*	0	NA
Vitamin D insufficiency	Liu	2022	12	high dose (> 400 IU/day) vs low dose (≤ 400 IU/day) or placebo	random-effect	RR	0.508	(0.384 ~ 0.673)*	96	NA
Vitamin D concentration	Liu	2022	25	high dose (> 400 IU/day) vs low dose (≤ 400 IU/day) or placebo	random-effect	MD	27.7	(20.5 ~ 34.9)*	98	NA
WB-BMC	Luo^a^	2022	2	high dose (> 400 IU/day) vs low dose (≤ 400 IU/day) or placebo	fixed-effect	MD	1.09	(-0.64 ~ 2.81)	0	NA
WB-BMD	Luo^a^	2022	2	high dose (> 400 IU/day) vs low dose (≤ 400 IU/day) or placebo	fixed-effect	MD	0.00	(0.00 ~ 0.00)	0	NA
WB-BA	Luo^a^	2022	2	high dose (> 400 IU/day) vs low dose (≤ 400 IU/day) or placebo	fixed-effect	MD	3.71	(-1.75 ~ 9.18)	0	NA

*MA* meta-analysis, *RR* relative risk, *MD* mean difference, *NA* not available, *: significant difference, *WB-BMC* whole body bone mineral content, *WB-BMD* whole body bone mineral density, *WB-BA* whole body bone area, *NICU* neonatal intensive care unit.

### Vitamin D supplementation for women with GDM

Two meta-analyses investigated that effects of vitamin D supplementation in pregnancy affected by GDM (Table 5) [17, 31]. It significantly reduced caesarean section rate (RR = 0.75; 95% CI = 0.63–0.89; number of studies = 9), hospitalization rate (RR = 0.13; 95% CI = 0.02–0.98; number of studies = 2), and preterm birth (OR = 0.37; 95% CI = 0.22–0.62; number of studies = 10) [17, 31].

**Table 5:** Summary of meta-analyses of randomized controlled trials on vitamin D supplementation for pregnant women with gestational diabetes mellitus and its impact on maternal and offspring health.

Outcomes	First author	Year	No. of articles in MA	Comparator (vitamin D supplementation or level)	Effects model	Metric of MA	Effect size	95% CI	*I*^2^ (%)	Publication bias
**Pregnant health**										
Preterm birth	Wu	2023	10	high dose (≥ 400 IU/day) vs placebo	fixed-effect	OR	0.37	(0.22 ~ 0.62)*	0	NA
Total cholesterol	Wu	2023	7	high dose (≥ 400 IU/day) vs placebo	random-effect	SMD	(-0.67)	(-1.19 ~ -0.14)*	85	NA
Low-density lipoprotein cholesterol	Wu	2023	7	high dose (≥ 400 IU/day) vs placebo	fixed-effect	SMD	(-0.49)	(-0.68 ~ -0.29)*	30	NA
Triglycerides	Wu	2023	6	high dose (≥ 400 IU/day) vs placebo	random-effect	SMD	(-0.59)	(-1.01 ~ -0.17)*	77	NA
High-density lipoprotein cholesterol	Wu	2023	8	high dose (≥ 400 IU/day) vs placebo	fixed-effect	SMD	0.41	(0.23 ~ 0.58)*	0	NA
25(OH)D	Wu	2023	9	high dose (≥ 400 IU/day) vs placebo	random-effect	SMD	4.07	(2.73 ~ 5.41)*	97	NA
HOMA-IR	Wang	2021	8	supplementation vs placebo or nothing	random-effect	MD	(-1.06 mmol/L)	(-1.40 ~ -0.72)*	74	NA
Cesarean section	Wang	2021	9	supplementation vs placebo or nothing	random-effect	RR	0.75	(0.63 ~ 0.89)*	43	NA
Hospitalization	Wang	2021	2	supplementation vs placebo or nothing	fixed-effect	RR	0.13	(0.02 ~ 0.98)*	0	NA
Postpartum hemorrhage	Wang	2021	2	supplementation vs placebo or nothing	fixed-effect	RR	0.47	(0.22 ~ 1.00)	0	NA
Fasting plasma glucose	Wang	2021	11	supplementation vs placebo or nothing	random-effect	MD	(-10.2 mg/dL)	(-13.43 ~ -6.96)*	80	NA
Fasting insulin level	Wang	2021	8	supplementation vs placebo or nothing	random-effect	MD	(-5.02 μIU/mL)	(-6.83 ~ -3.20)*	78	NA
**Infant health**										
Hyperbilirubinemia	Wu	2023	9	high dose (≥ 400 IU/day) vs placebo	fixed-effect	OR	0.38	(0.25 ~ 0.58)*	0	NA
Hospitalization	Wu	2023	4	high dose (≥ 400 IU/day) vs placebo	fixed-effect	OR	0.29	(0.16 ~ 0.53)*	0	NA
Giant children	Wang	2021	6	supplementation vs placebo or nothing	fixed-effect	RR	0.58	(0.38 ~ 0.89)*	0	NA
Fetal distress	Wang	2021	2	supplementation vs placebo or nothing	fixed-effect	RR	0.46	(0.24 ~ 0.90)*	0	NA
Polyhydramnios	Wang	2021	4	supplementation vs placebo or nothing	fixed-effect	RR	0.42	(0.24 ~ 0.72)*	0	NA
Hypoglycemia	Wang	2021	4	supplementation vs placebo or nothing	fixed-effect	RR	0.82	(0.52 ~ 1.29)	0	NA

*MA* meta-analysis, *OR* odds ratio, *RR* relative risk, *MD* mean difference, *SMD* standardized mean difference, *NA* not available; *significant difference; 25(OH)D 25-hydroxyvitamin D, *HOMA-IR* homeostasis model assessment of insulin resistance.

In terms of maternal biochemical changes, vitamin D supplementation (≥ 400 IU/day) significantly increased maternal 25-hydroxyvitamin D level (SMD = 4.07; 95% CI = 4.73 ~ 5.41; number of studies = 9) and high-density lipoprotein (SMD = 0.41; 95% CI = 0.23 ~ 0.58; number of studies = 8), and significantly decreased maternal fasting plasma glucose (MD = −0.2; 95% CI = −13.43 ~ −6.96; number of studies = 11), fasting insulin level (MD = −0.2; 95% CI = −6.83 ~ −3.20; number of studies = 8), HOMA-IR (MD = −1.06; 95% CI = −1.40 ~ −0.72; number of studies = 8), total cholesterol (SMD = −0.67; 95% CI = −1.19 ~ −0.14; number of studies = 8), low-density lipoprotein cholesterol (SMD = −0.49; 95% CI = −0.68 ~ −0.29; number of studies = 7), and triglycerides (SMD = −0.59; 95% CI = −1.01 ~ −0.17; number of studies = 18) compared to placebo or no supplementation [17, 31].

### Comparison between observational studies and RCTs

A low level of vitamin D during pregnancy significantly increased the risk of GDM and small-for-gestational-age infants, while vitamin D supplementation during pregnancy significantly reduced these risks. On the other hand, a low level of vitamin D during pregnancy significantly increased the risk of preterm birth, but vitamin D supplementation significantly reduced preterm birth risk only for women with GDM and not for those without it. This pattern was also observed in cases of pre-eclampsia. For birth length, a low level of vitamin D during pregnancy did not increase its risk. However, vitamin D supplementation during pregnancy significantly increased birth length.

## Discussion

### Principal findings

We reviewed 15 systematic reviews and meta-analyses involving 174,175 women. Our findings demonstrate that vitamin D deficiency during pregnancy is associated with adverse outcomes for both the mother and offspring. These outcomes include preterm birth, small-for-gestational-age/low birth weight babies, recurrent miscarriages, bacterial vaginosis, and GDM. Furthermore, vitamin D supplementation during pregnancy increases birth weight, reduces the risk of pre-eclampsia and miscarriage, and improves outcomes in women affected by GDM. However, the methodical quality of all meta-analyses was assessed as critically low.

### Comparison with existing literature

We found that having a low level of vitamin D during pregnancy significantly increases the risk of preterm birth and small-for-gestational-age infants, while high-quality evidence suggests vitamin D supplementation during pregnancy did not reduce these risks. As labor has features suggestive of an inflammatory response [35, 36], vitamin D deficiency during pregnancy could lead to increased levels of inflammatory cytokines [37] and potentially lead to preterm birth. Although vitamin D supplementation during pregnancy did not affect the risk of preterm birth, this may be due to the fact that the participants in the RCTs did not all have vitamin D deficiency, and thus the effect of vitamin D supplementation on preterm birth was not significant.

Our umbrella review showed that a low level of vitamin D ( < 50 nmol/L) in pregnancy significantly increased the risk of low birth weight (<2500 g). However, this evidence was considered as low because the result was obtained from observational studies and had high heterogeneity (*I*^2^ > 50%). We did not find any association between low level of vitamin D and birth length. On the other hand, a high dose of vitamin D supplementation in pregnancy significantly increased birth length but not increase birth wight. These results were obtained from RCTs, and their evidence was graded as “convincing”. Although heterogeneity was detected in the meta-analyses, the inclusion of a large number of studies (n > 20) strengthens the convincing nature of the results.

The incidence rate of recurrent miscarriage is about 1 ~ 2% [38]. It is critical to investigate the risk factors of recurrent miscarriage in order to determine the optimal treatment and prevent its recurrence. Our review demonstrated that vitamin D deficiency during pregnancy significantly increases the risk of recurrent miscarriage. Although the effect size is large (OR = 4.02) and the number of included articles is substantial (n = 14), there is high heterogeneity in the meta-analysis (*I*² = 82%) [23]. The assay method, age, and region were considered as sources of heterogeneity [23]. The evidence of this association was assessed as “Possible”. Nevertheless, no meta-analysis has looked into how vitamin D supplementation during pregnancy affects the chance of having recurrent miscarriage. We suggest that RCTs should be conducted to investigate this issue in the future.

Pregnant women with bacterial vaginosis are at risk for adverse outcomes such as low birth weight, preterm birth, fetal death, late miscarriage, and chorioamnionitis [39, 40]. Our review found that vitamin D deficiency during pregnancy is associated with a high risk of bacterial vaginosis. Although the meta-analysis included many studies (n = 14), there was high heterogeneity (*I*^2^ = 85%). To date, no meta-analysis has investigated the effect of vitamin D supplementation during pregnancy on the risk of bacterial vaginosis. There is one RCT which showed that vitamin D supplementation for women with vitamin D deficiency may reduce the risk of bacterial vaginosis [41]. Supplementing with vitamin D should reduce the risk of bacterial vaginosis in pregnant women with vitamin D deficiency. Further RCTs should be conducted to confirm this finding.

Pre-eclampsia is a common pregnancy complication affecting approximately 4 million pregnant women every year, and leading to over 70,000 maternal and 500,000 neonatal deaths globally [42, 43]. Our review showed that vitamin D supplementation during pregnancy may lower the risk of pre-eclampsia and, consequently, the risk of neonatal or fetal death. Pro-inflammatory cytokine secretion has been implicated in the pathogenesis of pre-eclampsia, and vitamin D supplementation could reduce the secretion through the inhabitation of Toll-like Receptor 4 monocyte expression [44].

We found low maternal vitamin D levels during pregnancy increased the risk of attention-deficit hyperactivity disorder, autism spectrum disorder, and mental development issues in offspring when compared to high maternal vitamin D levels. Since these results were obtained from observational studies, we deemed the evidence for association as only “possible” or “probable”. However, our finding is supported by the increase in gene expression related to the differentiation of dopaminergic neurons by vitamin D, which affects the neuronal development of the brain during the embryonic period [45]. Moreover, research indicates that children who experience deficiencies in vitamin D are more susceptible to developing attention-deficit hyperactivity disorder or autistic spectrum condition [46, 47], while vitamin D supplementation in children with attention-deficit hyperactivity disorder or autism spectrum disorder had improved cognitive function [48] or reduced severity [49], respectively.

Our comprehensive review found that vitamin D supplementation reduces the risk of GDM. However, vitamin D deficiency during pregnancy significantly increases the risk of GDM. This finding is supported by evidence from both observational studies and RCTs. In addition, a meta-analysis has reported vitamin D plus calcium supplementation can reduce the risk of type 2 diabetes mellitus, but only in people with glucose intolerance [50]. Therefore, vitamin D plays a vital role in preventing GDM. We also demonstrated that vitamin D supplementation for pregnant women with GDM reduced risks of polyhydramnios, preterm birth, Caesarean section, maternal hospitalization, macrosomia, newborn hyperbilirubinemia, and fetal distress, along with biochemical improvements. However, a letter to editor [51] pointed out some concerns with the meta-analysis conducted by Wang et al. [31]. In their update, vitamin D supplementation had no effect on the risk of preterm birth, Caesarean section, maternal hospitalization, macrosomia, or newborn hyperbilirubinemia [51]. However, some outcomes of this updated meta-analysis included only a few studies (n = 2 ~ 3), and was not formally published as an original article.

The RCTs used different doses of vitamin D supplementation to examine its impact on clinical outcomes. To assess the impact of dose on clinical outcomes, we included meta-analyses that compared two significantly different doses (2000 IU/day and 400 IU/day) as the cut-off points. These meta-analyses can help to investigate the effect of dose on clinical outcomes. Additionally, the meta-analyses also compared outcomes between vitamin D supplements (< 2000 IU/day) and placebo. These comparisons helped determine if a specific high dose was necessary to achieve significant improvement. According the current evidence, a very high dose of vitamin D supplement (> 2000 IU/day) only showed a significant improvement in the risk of GDM when compared with a low dose (≤ 2000 IU/day) (Table 3). Otherwise, a low dose of supplements (> 400 IU/day) could be used to improve several clinical outcomes, such as fetal or neonatal mortality, vitamin D insufficiency, and preterm birth (Table 3).

### Strengths and limitations

This study well-organized the current evidence of the topic investigating the impact vitamin D status during pregnancy on health-related outcomes. The newest and highest level of evidence of meta-analyses were included. In addition, we assessed the evidence of meta-analyses. However, this comprehensive review does have some limitations. First, some adverse clinical outcomes were reported to be associated with vitamin D deficiency in pregnancy based on the meta-analysis of observational studies. However, vitamin D supplementation in pregnancy could not prevent these adverse clinical outcomes based on the meta-analysis of RCTs. This may be due to the participants in the RCTs did not all have vitamin D deficiency. Second, some meta-analyses only included a few studies (n = 2 to 3), and the results may not be reliable. More primary studies should be conducted to confirm these results. Finally, vitamin D status in pregnancy was assessed at the time of blood sampling and only reflect the vitamin D status over a short period of time period. As daily vitamin D status in blood varies according to diet and sum exposure, long-term measures of vitamin D status would be more reliable, such as measurement from hair samples.

## Conclusion

Vitamin D deficiency is commonly found in pregnancy. This review highlights the adverse outcomes associated with low vitamin D levels during pregnancy and that vitamin D supplementation during pregnancy can reduce the risk of some adverse outcomes. Based on our review, it is suggested that pregnant women take a dose of > 400 IU/day of vitamin D supplementation during pregnancy to prevent certain adverse outcomes. However, there is conflicting evidence for the effectiveness of vitamin D supplementation in women with GDM. We recommend monitoring vitamin D levels during pregnancy, and supplementing with vitamin D for pregnant women who are deficient to prevent potential adverse outcomes.

### Supplementary information

Supplemental material

## Data Availability

All data used in this study come from the published articles listed in Table 1.
